# Trajectories of anxiety and depression among Chinese men who have sex with men on pre-exposure prophylaxis: a group-based trajectory model approach

**DOI:** 10.1186/s12889-024-17854-x

**Published:** 2024-02-03

**Authors:** Shuo Chen, Yan-Yan Zhu, Zhen-Xing Chu, Hui Zhou, Miao Liu, Yong-Jun Jiang, Qing-Hai Hu, for the China Real-World Oral Intake of PrEP (CROPrEP) Study Team

**Affiliations:** 1 State Key Laboratory for Diagnosis and Treatment of Infectious Diseases, NHC Key Laboratory of AIDS Immunology (China Medical University), National Clinical Research Centre for Laboratory Medicine, The First Hospital of China Medical University, Shenyang, China; 2 Key Laboratory of AIDS Immunology, Chinese Academy of Medical Sciences, Shenyang, China; 3 Key Laboratory of AIDS Immunology of Liaoning Province, Shenyang, China; 4 Collaborative Innovation Centre for Diagnosis and Treatment of Infectious Diseases, Hangzhou, China

**Keywords:** Men who have sex with men, Pre-exposure prophylaxis, Anxiety, Depression, Group-based trajectory model, Adherence

## Abstract

**Background:**

Anxiety and depression can influence adherence to Pre-exposure Prophylaxis (PrEP). However, there is limited research on the temporal dynamics of anxiety and depression among men who have sex with men (MSM) using PrEP.

**Methods:**

From December 2018 to November 2020, we administered the Hospital Anxiety and Depression Scale (HADS) to participants in the China Real-World Oral Intake of PrEP (CROPrEP) to measure their anxiety and depression levels. The group-based trajectory model (GBTM) depicted the dynamic changes of anxiety and depression scores over time.

**Results:**

A total of 1023 MSM were included, with 4523 follow-up assessments. The GBTM categorized the trajectories into three distinct patterns: consistently low (54.8% for anxiety, 60.7% for depression), consistently moderate (39.3% for anxiety, 31.4% for depression), and high but bell-shaped (5.9% for anxiety, 7.9% for depression). Higher anxiety levels were associated with being aged 18–30 years old, earning less than US$619 per month, female-identifying, adopting the bottom sexual role with men, and having two or more anal sex partners in the past three months; similarly, higher depression levels correlated with a monthly income under US$619, female-identifying, sexual behavior as bottom and a positive syphilis at baseline. PrEP adherence was notably lower in the high but bell-shaped anxiety and depression group compared to the other groups, particularly at the 12th-month follow-up.

**Conclusions:**

Close monitoring of anxiety and depression levels in MSM on PrEP is crucial. Provision of targeted mental health support is essential to enhance PrEP effectiveness.

**Supplementary Information:**

The online version contains supplementary material available at 10.1186/s12889-024-17854-x.

## Background

The HIV/AIDS epidemic persists, with among men who have sex with men (MSM) disproportionately affected. Globally, as of 2022, an estimated 39 million people were living with HIV, with MSM prevalence at 7.7%, markedly exceeding the general population's rate of 0.7% [1]. In China, HIV/AIDS cases reached 1.2 million by 2022’s end, with MSM prevalence surpassing 7%, significantly higher than the 0.1% in the broader population [2]. Pre-exposure prophylaxis (PrEP), an innovative biomedical intervention employing antiviral drugs, has proven highly effective in preventing HIV [3], slashing the risk by about 99% during sexual exposure [4]. The China Real-World Oral Intake of PrEP (CROPrEP) study observed no new HIV infections among highly adherent MSM over a 12-month period [5]. Adherence plays a crucial role in ensuring the effectiveness of PrEP, which can be influenced by various factors, including stigma, discrimination, side effects, and compromised mental well-being [6].

Due to their unique social identity, MSM often encounter different forms of stigma, leading to emotional distress, social isolation, and physiological reactivity [7]. Individual, interpersonal (intimate partner violence and low social support), community-level and structural (gender inequality) factors at different levels can contribute to elevated risk of depression and HIV acquisition among MSM [8]. A meta-analysis revealed that the overall prevalence of anxiety symptoms among Chinese MSM was 32.2% [9]. Several studies have established a connection between anxiety, depression, and reduced PrEP adherence [10–12]. A study in China also found that psychological factors had significant effects on the willingness to use and adherence to PrEP [13]. However, these studies mainly focus on the relationship between anxiety/depression scores at one or multiple time points and PrEP adherence, overlooking the fluctuations in anxiety/depression scores experienced by study participants during PrEP use.

The Group-based trajectory model (GBTM) aims to identify distinct clusters of individual trajectories within a population and analyze the characteristics of individuals within each group [14]. In the HIV Prevention Trials Network 082, GBTM was used to identify a significant inverse relationship between elevated depressive symptoms and PrEP use during the follow-up period among adolescent girls [15]. Wu et al. used GBTM and observed that the proportions of MSM in the low, moderate, and high anxiety groups were 32.56%, 56.12%, and 11.32%, respectively [16]. Li et al. categorized young and middle-aged MSM in Beijing into three groups based on trajectories of depressive symptoms after new HIV-diagnosis [17]. Despite the positive outcomes of GBTM in assessing the influence of depressive symptom trajectories on PrEP adherence, few studies have explored this topic among MSM [18]. Therefore, there is an urgent need to understand the fluctuations in anxiety and depression among MSM on PrEP, as well as the factors that contribute to varying levels of anxiety and depression.

During the implementation of the CROPrEP project, we collected data on anxiety and depression scores, as well as PrEP adherence [5]. This study employed GBTM to examine the fluctuations in anxiety and depression scores among MSM using PrEP, investigated the factors that influence the levels of anxiety and depression, and assessed the characteristics in PrEP adherence across different subgroups.

## Methods

### Study design and participants

The CROPrEP project was a multicentre study conducted in four metropolitan cities (Beijing, Shenyang, Shenzhen, and Chongqing) in China from December 11, 2018, to November 30, 2020 [5, 19]. Eligible MSM could choose between daily PrEP (D-PrEP) and event-driven PrEP (ED-PrEP), based on their personal preference. Participants had the flexibility to switch between regimens during the study. D-PrEP involved taking one pill every 24 h, while ED-PrEP involved taking two pills within 2 to 24 h before sexual intercourse (or one pill if the last dose was taken 1–6 days ago), followed by one pill every 24 h during sexual activity, including after the last sexual encounter, and one final pill approximately 24 h later. Participants were followed up for 12 months with six clinic visits at designated centres, including the baseline and follow-up visits at 1, 3, 6, 9, and 12 months. Both D-PrEP and ED-PrEP users completed a weekly diary on their smartphones to track pill intake and sexual activities.

### Data collection

A dedicated survey platform (https://jinshuju.net/) was used to collect self-administered questionnaires. The survey comprised sociodemographic information (age, education, monthly income, gender identity, marital status), sexual roles with men, number of anal sex partners, and condomless anal intercourse (CAI) in the past three months. HIV knowledge was measured using the 8-item HIV Knowledge Questionnaire, widely used in China with good validity [20]. Respondents answering all eight questions correctly were considered to have good awareness. During the follow-up visits, participants underwent HIV testing (antigen–antibody testing and HIV-RNA testing). Syphilis serological screening at baseline was performed using the rapid plasma regain (RPR) test, with positive results confirmed by the Treponema pallidum particle agglutination (TPPA) assay. Individuals testing positive on both RPR and TPPA were considered to have active infections.

The Hospital Anxiety and Depression Scale (HADS) was used to determine participants' anxiety and depression scores. It included seven items for assessing depression and seven items for assessing anxiety, each scored from zero to three. A score of eight or higher on either scale indicated the presence of anxiety or depression [21]. The Cronbach’s alpha coefficients for the overall HADS, anxiety, and depression subscale were 0.879, 0.806, and 0.806, respectively, suggesting good internal consistency within the measures [22].

Adherence scores for D-PrEP were calculated based on the ratio of consumed pills to the theoretical number of pills to be consumed, representing the number of days between medication pickups. For ED-PrEP, adherence scores were determined by the coverage of anal intercourse by PrEP. The coverage of anal intercourse by PrEP was calculated based on the ratio of “sex-days” (days when anal intercourse with one or more men occurred) to the instances PrEP was correctly taken. A correct PrEP intake involved administering the appropriate dose before, during, and after the days of sexual activity. The correct dose before required at least two pills taken on days X (a sex day) or X-1 (the day before); or one pill on X or X-1 if a pill was taken between day X-6 and X-1. The last situation applied when an individual was on D-PrEP, or if the interval between two episodes of ED-PrEP was less than a week. A correct dose during and after involved taking at least one pill on days X, X + 1 and X + 2. Adherence was achieved in both groups when adherence scores exceeded 0.9. Information on sexual activity and pill intake was obtained from weekly diaries maintained by participants. To ensure accuracy, staff cross-checked self-reported intakes against pill dispensing records and counts during each follow-up clinic visit [5].

### Group-based trajectory model

The GBTM was employed to evaluate the changes in scores for anxiety and depression from baseline to each follow-up among the participants. As a semi-parametric model designed for longitudinal data, GBTM assumed a discrete distribution within the population. This approach identified subgroups or classes of homogeneous individuals characterized by similar trajectories [23]. GBTM was fitted sequentially for anxiety and depression, with 1 to 5 subgroups included in each model. The quality of each model’s fit was evaluated using the Bayesian Information Criterion (BIC) and the Average Posterior Probability (AvePP). A BIC value closed to 0, along with an AvePP of over 0.7, indicated a good fit.

### Statistical analysis

Multiple imputation was used for missing outcome data, incorporating all baseline characteristics (age, educational level, monthly income, psychological gender identity, marital status, sexual role with men, No. of anal sex partners in the past three months, episodes of CAI in the past three months, HIV knowledge, and syphilis infection at baseline), HADS scores and adherence scores at all time points, and stratification variables to generate five imputed data sets. Frequencies and rates represented the categorical data. Univariate analysis was performed using the χ^2^ test, and variables with *P* < 0.150 were included in the multivariate ordered logistic regression. Kruskal–Wallis test was used to examine differences in adherence scores at different follow-up points. The Bonferroni-corrected χ^2^ test was used to assess the association between different anxiety/depression trajectories and adherence scores trajectories. All variables were statistically significant at P < 0.050. All statistical analyses used SPSS 27.0 (SPSS, IBM Inc.), and the lcmm package in R 4.3.1 (The R Foundation for Statistical Computing) was used to construct GBTM. GraphPad Prism 9.5.0 (GraphPad Software, LLC) was used to create graphs.

## Results

### Participant characteristics

**Fig. 1:**
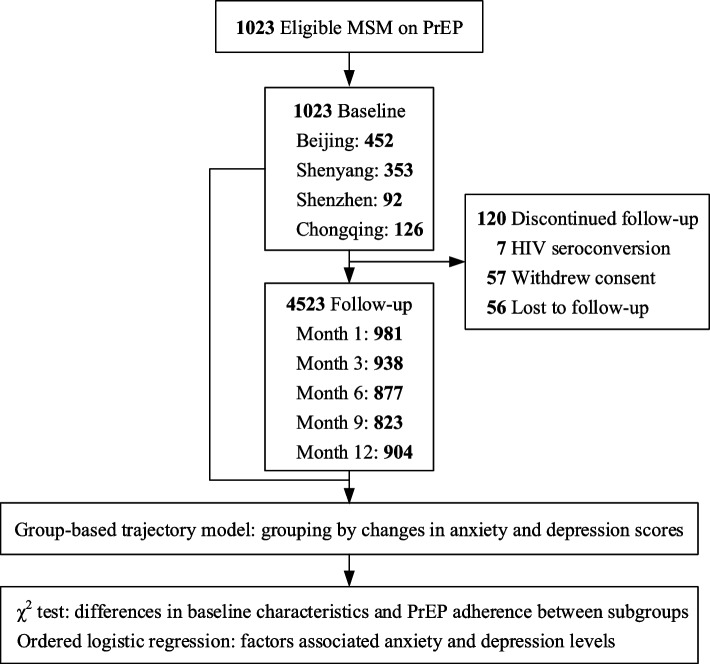
Study profile

The study commenced with 1,023 eligible MSM, comprising 353 (34.5%) from Shenyang, 452 (44.2%) from Beijing, 92 (9.0%) from Shenzhen, and 126 (12.3%) from Chongqing. During the 12-month observation period, a total of 4,523 follow-up entries were documented. Nevertheless, 120 (11.7%) participants discontinued their follow-up due to reasons including HIV seroconversion, withdrawal consent, or lost to follow-up. Most participants were 31–50 years old (59.7%) and had a monthly income exceeding US$619 (67.2%). Over half were single (54.5%) and a significant proportion reported versatile sexual behaviour (43.8%). Notably, 82.3% and 77.7% of participants reported having two or more sexual partners and engaged in CAI in the past three months, respectively. Moreover, the prevalence of active syphilis infection at baseline was 10.0% (Table 1 and Fig. 1).

**Table 1:** Baseline characteristics of the participants (*n* = 1023)

Characteristics	*n* (%)
Age, median (IQR), years	33 (29–40)
Age, years	
18–30	336 (32.8)
31–50	611 (59.7)
> 50	76 (7.4)
Educational level	
High school or less	192 (18.8)
College and greater	831 (81.2)
Monthly income, US$	
< 619	336 (32.8)
≥ 619	687 (67.2)
Psychological gender identity	
Male	998 (97.6)
Female	25 (2.4)
Marital status	
Single	558 (54.5)
Married/cohabitation	439 (42.9)
Separated, divorced, or widowed	26 (2.5)
Sexual role with men	
Top	287 (28.1)
Bottom	281 (27.5)
Versatile	448 (43.8)
Oral	7 (0.7)
No. of anal sex partners in the past three months	
< 2	181 (17.7)
≥ 2	842 (82.3)
Episodes of CAI in the past three months	795 (77.7)
HIV knowledge	728 (71.2)
Baseline syphilis positivity	102 (10.0)

*Abbreviations*: *IQR* interquartile range, *CAI* condomless anal intercourse

### Group-based trajectory modeling of anxiety and depression

**Fig. 2:**
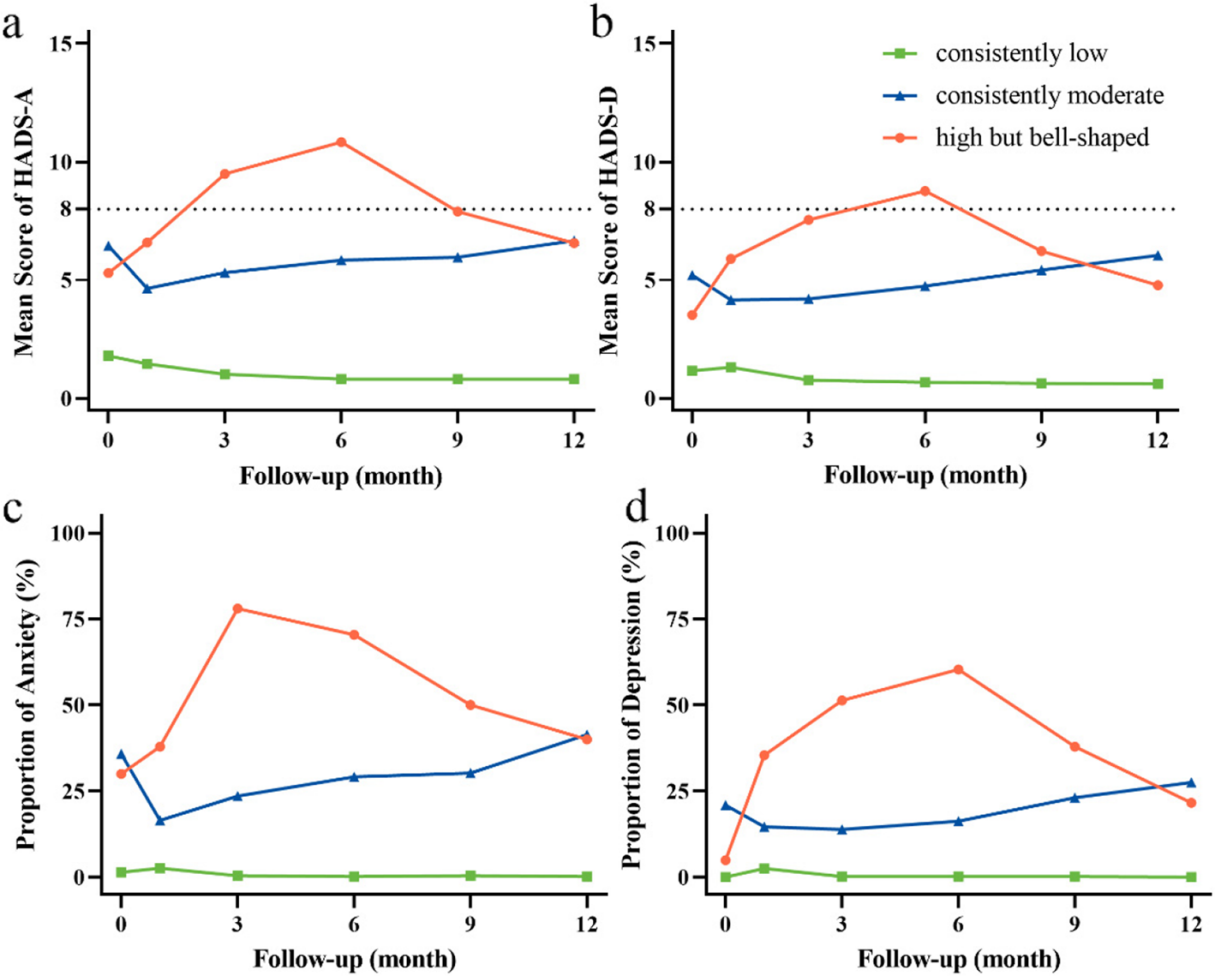
Trajectory profiles for anxiety and depression. **a** Mean scores of HADS-A in different anxiety trajectories; (**b**) Mean scores of HADS-D in different depression trajectories; (**c**) Proportions of anxiety in different anxiety trajectories, where the Y-axis represents the proportion of people with anxiety scores ≥ 8; (**d**) Proportions of depression in different depression trajectories, where the Y-axis represents the proportion of people with depression scores ≥ 8. Abbreviations: HADS-A, Hospital Anxiety and Depression Scale-Anxiety; HADS-D, Hospital Anxiety and Depression Scale-Depression

GBTM analyzed for anxiety and depression identified trajectories ranging from one to five (Supplementary material: Table S1). The three-trajectory solution provided the optimal model fit, as evidenced by BIC values approaching zero and AvePP exceeding 0.7. As shown in Fig. 2, these trajectories (consistently low, consistently moderate and high but bell-shaped) chart the progression of anxiety and depression scores over time. The majority of cases were consistently low group, accounting for 54.8% (561/1023) in terms of anxiety trajectories and 60.7% (621/1023) of depression trajectories, featuring minimal score fluctuations (anxiety: 0.81–1.80; depression: 0.61–1.31) and low proportions (anxiety: 0.2%-2.6%; depression: 0–2.5%). The consistently moderate group showed an initial decrease, followed by a gradual rise throughout the study period (anxiety scores: 4.64–6.67; depression scores: 4.16–6.04; anxiety proportions: 16.5%-41.4%; depression proportions: 13.8%-27.5%). Conversely, the high but bell-shaped group peaked mid-study before subsiding (anxiety scores: 5.30–10.83; depression scores: 3.52–8.76; with anxiety proportions peaking at 78.0% in the third month and depression proportions reaching up to 60.3%).

### Characteristics with different levels of anxiety and depression

Table 2 demonstrated the association of sociodemographic and behavioral characteristics on levels of anxiety and depression. Individuals aged 18–30, with a monthly income below US$619, identifying as female, single, and playing the bottom role in male-to-male sex had a higher prevalence in the high but bell-shaped anxiety group. Furthermore, individuals earning less than US$619 monthly, identifying as female, and who engaged in CAI in the past three months had a higher prevalence in the high but bell-shaped depression group.

**Table 2:** Comparison of characteristics among participants in different trajectory groups of anxiety and depression (*n* = 1023)

Characteristics	Anxiety				Depression			
	**Consistently low (*n* = 561)**	**Consistently moderate (*n* = 402)**	**High but bell-shaped (*n* = 60)**	***P***	**Consistently low (*n* = 621)**	**Consistently moderate (*n* = 321)**	**High but bell-shaped (*n* = 81)**	***P***
Age, years				**0.020**				0.769
18–30	170 (30.3)	138 (34.3)	28 (46.7)		195 (31.4)	112 (34.9)	29 (35.8)	
31–50	339 (60.4)	243 (60.4)	29 (48.3)		380 (61.2)	184 (57.3)	47 (58.0)	
> 50	52 (9.3)	21 (5.3)	3 (5.0)		46 (7.4)	25 (7.8)	5 (6.2)	
Educational level				0.530				**0.058**
High school or less	110 (19.6)	69 (17.2)	13 (21.7)		102 (16.4)	72 (22.4)	18 (22.2)	
College and greater	451 (80.4)	333 (82.8)	47 (78.3)		519 (83.6)	249 (77.6)	63 (77.8)	
Monthly income, US$				**0.023**				** < 0.001**
< 619	164 (29.2)	148 (36.8)	24 (40.0)		176 (28.3)	126 (39.3)	34 (42.0)	
≥ 619	397 (70.8)	254 (63.2)	36 (60.0)		445 (71.7)	195 (60.7)	47 (58.0)	
Psychological gender identity				**0.007**				**0.013**
Male	553 (98.6)	390 (97.0)	55 (91.7)		612 (98.6)	310 (96.6)	76 (93.8)	
Female	8 (1.4)	12 (3.0)	5 (8.3)		9 (1.4)	11 (3.4)	5 (6.2)	
Marital status				**0.045**				0.182
Single	285 (50.8)	233 (58.0)	40 (66.7)		326 (52.5)	190 (59.2)	42 (51.9)	
Married/cohabitation	258 (46.0)	161 (40.0)	20 (33.3)		480 (45.1)	124 (38.6)	35 (43.2)	
Separated, divorced, or widowed	18 (3.2)	8 (2.0)	0 (0.0)		15 (2.4)	7 (2.2)	4 (4.9)	
Sexual role with men				**0.004**				**0.102**
Top	182 (32.4)	90 (22.4)	15 (25.0)		187 (30.1)	86 (26.8)	14 (17.2)	
Bottom	129 (23.0)	131 (32.6)	21 (35.0)		156 (25.1)	100 (31.2)	25 (30.9)	
Versatile	246 (43.9)	178 (44.3)	24 (40.0)		274 (44.1)	132 (41.1)	42 (51.9)	
Oral	4 (0.7)	3 (0.7)	0 (0.0)		4 (0.7)	3 (0.9)	0 (0.0)	
No. of anal sex partners in the past three months				**0.103**				0.175
< 2	112 (20.0)	59 (14.7)	10 (16.7)		121 (19.5)	48 (15.0)	12 (14.8)	
≥ 2	449 (80.0)	343 (85.3)	50 (83.3)		500 (80.5)	273 (85.0)	69 (85.2)	
CAI in the past three months	445 (79.3)	301 (74.9)	49 (81.7)	0.197	493 (79.4)	235 (73.2)	67 (82.7)	**0.051**
HIV knowledge	403 (71.8)	285 (70.9)	40 (66.7)	0.695	459 (73.9)	218 (67.9)	51 (63.0)	**0.037**
Baseline syphilis positivity	48 (8.6)	47 (11.8)	7 (11.7)	0.252	50 (8.1)	42 (13.2)	10 (12.5)	**0.036**

*Abbreviations*: *CAI* condomless anal intercourse

The multivariate analysis included variables with a *P*-value < 0.150, using the consistently low group as the reference in the ordered logistic regression model. The analysis indicated that individuals aged 18–30 years old (vs. > 50, adjusted odds ratio [aOR] = 1.77, 95% confidence interval [CI]: 1.02–3.07), those with a monthly income below US$619 (vs. ≥ 619, aOR = 1.41, 95%CI: 1.06–1.86), female-identifying individuals (vs. male, aOR = 2.69, 95%CI: 1.23–5.90), those who played the bottom role in male-to-male sex (vs. top, aOR = 1.78, 95%CI: 1.27–2.50), and those who had two or more anal sex partners in the past three months (vs. < 2, aOR = 1.44, 95%CI: 1.03–2.01) were more likely to experience higher anxiety levels (all *P* < 0.050). Additionally, individuals with a monthly income below US$619 (vs. ≥ 619, aOR = 1.50, 95%CI: 1.14–1.96), female-identifying individuals (vs. male, aOR = 2.64, 95%CI: 1.22–5.74), those who played the bottom role in male-to-male sex (vs. top, aOR = 1.50, 95%CI: 1.07–2.11), and those with baseline syphilis positivity (aOR = 1.60, 95%CI: 1.07–2.39) were more likely to have higher depression levels (all *P* < 0.050) (Table 3).

**Table 3:** Multiple ordered logistic regression analysis for the associated factors of anxiety and depression

Characteristics	Anxiety			Depression	
	**aOR (95% CI)**		***P***	**aOR (95% CI)**	***P***
Age, years (vs. > 50)					
18–30	1.77 (1.02–3.07)		**0.041**	NA	NA
31–50	1.64 (0.97–2.78)		0.064	NA	NA
Educational level (vs. High school or less)					
College and greater	NA		NA	0.83 (0.59–1.16)	0.267
Monthly income, US$ (vs. ≥ 619)					
< 619	1.41 (1.06–1.86)		**0.017**	1.50 (1.14–1.96)	**0.003**
Psychological gender identity (vs. Male)					
Female	2.69 (1.23–5.90)		**0.013**	2.64 (1.22–5.74)	**0.014**
Marital status (vs. Single)					
Married/cohabitation	0.83 (0.64–1.07)		0.145	NA	NA
Separated, divorced, or widowed	0.51 (0.21–1.24)		0.137	NA	NA
Sexual role with men (vs. Top)					
Bottom	1.78 (1.27–2.50)		**0.001**	1.50 (1.07–2.11)	**0.020**
Versatile	1.32 (0.97–1.79)		0.079	1.21 (0.89–1.65)	0.224
Oral	1.02 (0.21–4.88)		0.983	1.07 (0.23–4.99)	0.932
No. of anal sex partners in the past three months (vs. < 2)					
≥ 2	1.44 (1.03–2.01)		**0.035**	NA	NA
CAI in the past three months (vs. No)					
Yes	NA		NA	0.79 (0.58–1.07)	0.126
HIV knowledge (vs. No)					
Yes	NA		NA	0.76 (0.58–1.01)	0.056
Baseline syphilis positivity (vs. No)					
Yes		NA	NA	1.60 (1.07–2.39)	**0.022**

*Abbreviations*: *CI* confidence interval, *NA* not applicable, *CAI* condomless anal intercourse

### Proportion of PrEP adherence at different levels of anxiety and depression

**Fig. 3:**
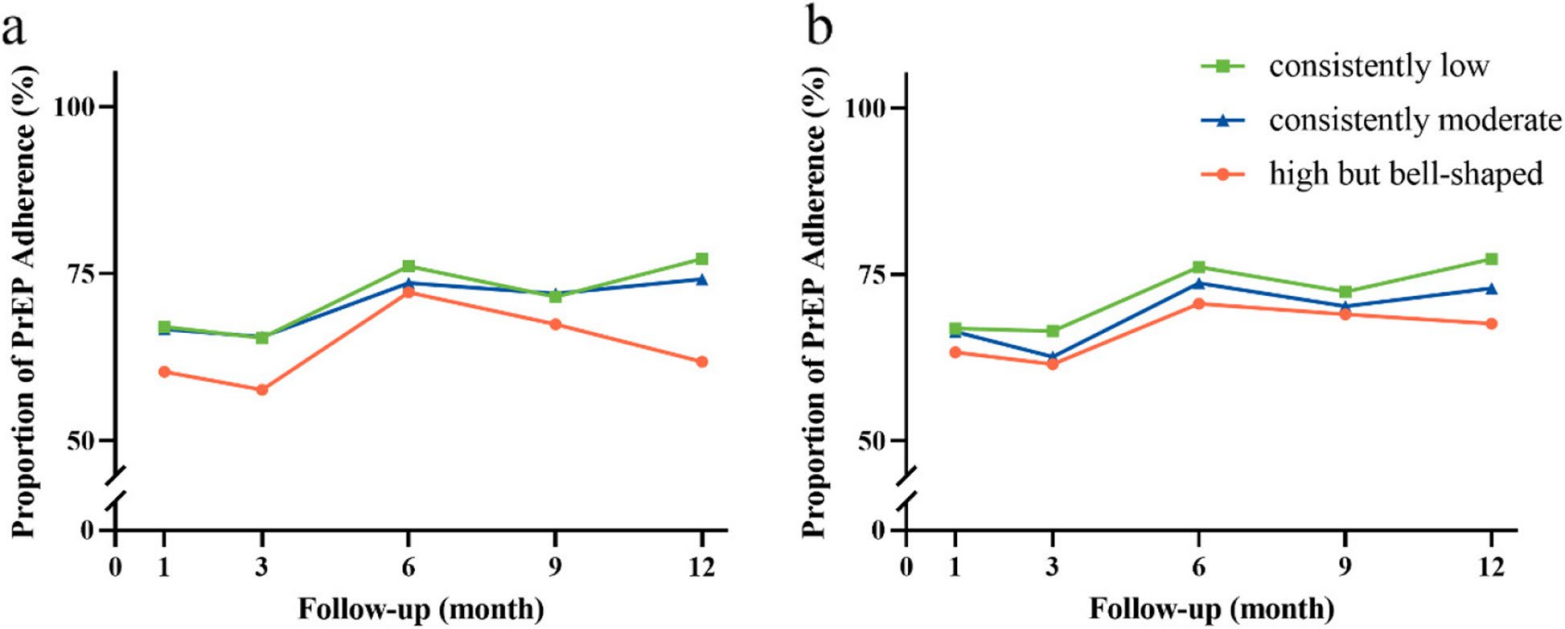
Proportion of PrEP adherence with different anxiety and depression levels. **a** Proportion of PrEP adherence with different anxiety levels, where the Y-axis represents the proportion of adherence scores > 0.9; (**b**) Proportion of PrEP adherence with different depression levels, where the Y-axis represents the proportion of adherence scores > 0.9

Figure 3 illustrated that the high but bell-shaped group of anxiety demonstrated lower proportion of PrEP adherence compared to the other two groups of anxiety, particularly in the 12th month (*P* = 0.038). However, there were no significant differences in the median adherence scores among different levels of anxiety at various follow-up points (all *P* > 0.050). Similarly, no significant differences were observed in the proportion of PrEP adherence and median adherence scores among different levels of depression (all *P* > 0.050) (Supplementary material: Table S2 and S4).

The GBTM method was used to categorize PrEP adherence, revealing two distinct trajectories: high adherence (median adherence scores of 1.00) and low adherence (median adherence scores ranging from 0.51 to 0.95) (Supplementary material: Table S5 and Fig. S1). No significant differences were observed in the proportion of distinct adherence trajectories among various anxiety and depression trajectories (Supplementary material: Table S6). Out of the seven newly identified HIV infections during the follow-up period, the majority were observed in the consistently low and consistently moderate anxiety and depression subgroups (Supplementary material: Table S7).

## Discussion

In this study, GBTM delineated three distinct anxiety and depression trajectories among MSM using PrEP: consistently low, consistently moderate, and high but bell-shaped. Higher anxiety and depression levels were associated with monthly income, psychological gender identity, and sexual role with men. Notably, increased anxiety undermined PrEP adherence, while depression did not exert a measurable impact. To the best of our knowledge, few studies identify varied trajectories of anxiety and depression among MSM using PrEP in China. This study indicates that when promoting PrEP among MSM, attention should be paid to their mental health during the medication process to enhance medication adherence and prevention efficacy.

In a novel application of GBTM, this research assessed mental health fluctuations among MSM on PrEP. A prior study in China also utilized the GBTM to classify MSM into low, moderate, and high anxiety groups, as well as low and high depression groups based on the trajectory analysis [16]. Our research indicated that participants in the high but bell-shaped anxiety and depression group tend to exhibit an increasing trend in their mean scores from the beginning of the study to the sixth month, which then decreases from six to twelfth months. This could be attributed to initial concerns about PrEP efficacy and side effects, resulting in increased levels of anxiety and depression. However, after a period of use, the realization of PrEP’s role in reducing the risk of HIV infection leads to a decrease in these levels [24]. Contrary to our findings, another study in Amsterdam found no significant temporal changes in the proportion of individuals diagnosed with anxiety or depressive mood disorders across both regimens [25]. However, it should be noted that this study assessed anxiety and depression on an annual basis, potentially overlooking short-term fluctuations. We found that, within the high but bell-shaped anxiety and depression group, up to 78.0% and 60.3% of individuals may exhibit symptoms of anxiety and depression, respectively. Therefore, future research should not only focus on behavioral changes among MSM on PrEP but also regularly monitor their mental health, ensuring appropriate monitoring intervals.

Several factors influence the levels of anxiety and depression among MSM using PrEP. A comparative study conducted in Western China found that the high prevalence of anxiety and depression was linked to factors such as younger age and lower monthly income [26]. This aligns with the results of our research. MSM and psychologically as female are more susceptible to stigma and violence because of their distinct social identity. This increased vulnerability can contribute to mental health issues such as anxiety and depression [27–29]. Our findings suggest that playing the bottom role in male-to-male sex is associated with elevated levels of anxiety. A plausible explanation could be that MSM who exclusively assume a bottom role in sexual activities often adopt a passive and submissive position, which may not fulfill their psychological and physiological needs. This can lead to long-term frustrations that manifest as symptoms of anxiety and depression [30]. Wu et al. found that MSM with syphilis were more likely to have a higher physical depression score [31]. Therefore, to maximize the preventive effectiveness of PrEP among MSM, it is essential to prioritize attention on economically disadvantaged, young MSM who identify as female, play the bottom role in male-to-male sex, have multiple sexual partners, and test positive for syphilis.

In our research, we discovered a correlation between elevated levels of anxiety symptoms and decreased PrEP adherence. Previous studies have also evaluated the negative effect of symptoms of anxiety and depression on PrEP adherence [10, 25, 32–34]. However, these investigations have predominantly overlooked the fluctuations in anxiety and depression among MSM throughout the entire PrEP utilization process. Young et al. discovered that 61.5% of participants exhibiting anxiety had protective tenofovir levels, in contrast to 81.8% of other participants [12]. Conversely, an investigation among MSM in New York City revealed no significant correlation between depressive symptoms and PrEP adherence [35]. In our research, it was observed that only elevated levels of anxiety at the 12th-month follow-up were correlated with decreased PrEP adherence, potentially related to the lockdown imposed during the COVID-19 pandemic. A study in France revealed that 58.8% of MSM reported discontinuing PrEP during the COVID-19 lockdown, and 15.4% were not utilizing PrEP at the time of the survey [36]. It is imperative to closely monitor symptoms of anxiety and depression among MSM throughout the PrEP utilization process to enhance adherence and consequently improve its effectiveness.

There are consistent challenges in adhering to PrEP as prescribed. Four HIV seroconverters had adherence scores below 0.9, indicating poor adherence in our study. The adherence scores of the other three seroconverters at their last negative follow-up visits were all above 0.9. However, the duration between last negative result and initial positive result was relatively long, exceeding two months. The above results indicate the importance of PrEP adherence in preventing HIV infection. A recent study showed that short message service reminders were ineffective in promoting PrEP adherence among young Kenyan women [37]. Therefore, it is crucial to evaluate and implement additional innovative interventions, such as real-time monitoring and just-in-time intervention, to support PrEP use among MSM [38].

This study acknowledges several limitations. Primarily, apart from a lower proportion of PrEP adherence observed in the high but bell-shaped group of anxiety in the 12th month, no significant differences in PrEP adherence were found across different levels of anxiety and depression in other analyses. This might be attributed to the limitations of the HADS scale, which only measures anxiety and depression levels in the past month and does not reflect the anxiety and depression experienced by MSM over the course of the past three months while taking PrEP. Secondly, due to attrition during the follow-up process, the accuracy of GBTM results may be impacted. However, the attrition rate in this study is relatively low (11.7%, 120/1023), and the GBTM demonstrates resilience to data lost to follow-up, bolstering the credibility of the results. Thirdly, compared to other trajectory model studies, the BIC in this article is relatively high, which may be attributed to the skewed distribution of HADS scores. However, in addition to BIC, we also utilized AvePP for trajectory model selection, enhancing the credibility of the results. Fourth, adherence was evaluated based on self-reported questionnaires. Participants may underreport information about sexual behavior due to societal stigma. However, we cross-verified self-reported medication intake with pill dispensing records and pill counts at each follow-up to enhance adherence accuracy. Lastly, given that this study was conducted within a PrEP demonstration project, the PrEP adherence outcomes may not be extrapolatable to real-world scenarios where adherence support structures may not be as robust. Future studies conducted after the broader promotion of PrEP may yield improved results.

## Conclusion

Our study findings demonstrate that MSM in China experience diverse levels of anxiety and depression while using PrEP. Factors including age, monthly income, gender identity, sexual roles with male partners, number of anal sex partners, and syphilis infection are associated with these levels of anxiety and depression. Higher levels of anxiety and depression potentially lead to decreased PrEP adherence. Therefore, it is crucial to closely monitor fluctuations in anxiety and depression levels among MSM using PrEP and provide suitable mental health support to optimize the effectiveness of PrEP.

### Supplementary Information

**Additional file 1: Table S1.** Fitting models for various anxiety and depression trajectories. **Table S2.** Proportion of PrEP adherence at different follow-up points for each trajectory (*n*=1023). **Table S3.** China Real-World Oral Intake of PrEP (CROPrEP) study team. **Table S4.** Scores of PrEP adherence at different follow-up for each anxiety and depression trajectory. **Table S5.** Fitting models for various PrEP adherence trajectories. **Table S6.** Association between different trajectories of anxiety and depression and trajectories of PrEP adherence scores. **Table S7.** Participants initiating PrEP with HIV seroconversion during the study period.

## Data Availability

The datasets generated and/or analyzed during the current study are not publicly available due to confidentiality policies but are available from the corresponding author on reasonable request.

## Abbreviations

AvePP: Average Posterior Probability
BIC: Bayesian Information Criterion
CAI: Condomless anal intercourse
CI: Confidence interval
CROPrEP: China Real-World Oral Intake of PrEP
D-PrEP: Daily PrEP
ED-PrEP: Event-driven PrEP
GBTM: Group-based trajectory model
HADS: Hospital Anxiety and Depression Scale
HADS-A: Hospital Anxiety and Depression Scale-Anxiety
HADS-D: Hospital Anxiety and Depression Scale-Depression
MSM: Men who have sex with men
NA: Not applicable
PLHIV: People living with HIV
PrEP: Pre-exposure prophylaxis
RPR: Rapid plasma regain
TPPA: Treponema pallidum particle agglutination
