# Supplementary material for: Trajectories of anxiety and depression among Chinese men who have sex with men on pre-exposure prophylaxis: a group-based trajectory model approach
Source: BMC Public Health. 2024 Feb 3;24:365. doi: 10.1186/s12889-024-17854-x (PMC10837939; doi:10.1186/s12889-024-17854-x)
Supplement: Supplementary file 1 — Additional file 1: Table S1. Fitting models for various anxiety and depression trajectories. Table S2. Proportion of PrEP adherence at different follow-up points for each trajectory (n=1023). Table S3. China Real-World Oral Intake of PrEP (CROPrEP) study team. Table S4. Scores of PrEP adherence at different follow-up for each anxiety and depression trajectory. Table S5. Fitting models for various PrEP adherence trajectories. Table S6. Association between different trajectories of anxiety and depression and trajectories of PrEP adherence scores. Table S7. Participants initiating PrEP with HIV seroconversion during the study period. [file 12889_2024_17854_MOESM1_ESM.docx]

**Supplementary Materials**

Supplement to: Shuo Chen, Yan-Yan Zhu, Zhen-Xing Chu, Hui Zhou, Miao Liu, Yong-Jun Jiang, Qing-Hai Hu, for the China Real-World Oral Intake of PrEP (CROPrEP) Study Team. **Trajectories of Anxiety and Depression among Chinese Men Who Have Sex with Men on Pre-exposure Prophylaxis: A Group-Based Trajectory Model Approach**

**Table S1** Fitting models for various anxiety and depression trajectories

| **Variable** | **Subgroup number** | **BIC** | **AvePP (95% CI)** | | | | |
| --- | --- | --- | --- | --- | --- | --- | --- |
|  |  |  | **1** | **2** | **3** | **4** | **5** |
| Anxiety | 1 | 27501.55 | 1.00  (1.00-1.00) | NA | NA | NA | NA |
|  | 2 | 26999.89 | 0.89  (0.50-0.97) | 0.95  (0.50-1.00) | NA | NA | NA |
|  | 3 | 26975.63 | 0.89  (0.39-0.89) | 0.76  (0.41-1.00) | 0.86  (0.41-1.00) | NA | NA |
|  | 4 | 26979.36 | 0.89  (0.38-0.98) | 0.86  (0.51-1.00) | 0.87  (0.43-1.00) | 0.77  (0.51-1.00) | NA |
|  | 5 | 26998.81 | 0.73  (0.38-0.98) | 0.69  (0.45-1.00) | 0.91  (0.45-0.99) | 0.74  (0.42-1.00) | 0.78  (0.39-0.98) |
| Depression | 1 | 26393.69 | 1.00  (1.00-1.00) | NA | NA | NA | NA |
|  | 2 | 25759.14 | 0.92  (0.50-0.97) | 0.94  (0.51-1.00) | NA | NA | NA |
|  | 3 | 25709.24 | 0.92  (0.36-0.98) | 0.77  (0.43-1.00) | 0.84  (0.44-1.00) | NA | NA |
|  | 4 | 25743.89 | 0.59  (0.34-0.62) | 0.75  (0.30-1.00) | 0.00  (0.00-0.00) | 0.82  (0.34-1.00) | NA |
|  | 5 | 25732.91 | 0.00  (0.00-0.00) | 0.78  (0.34-1.00) | 0.74  (0.40-1.00) | 0.66  (0.36-0.70) | 0.69  (0.34-0.93) |

Abbreviations: BIC, Bayesian Information Criterion; AvePP, Average Posterior Probability; CI, Confidence Interval; NA, not applicable

**Table S2** Proportion of PrEP adherence at different follow-up points for each trajectory (n=1023)

| **Follow-up points** | **Anxiety (n, %)** | | | |  | **Depression (n, %)** | | | |
| --- | --- | --- | --- | --- | --- | --- | --- | --- | --- |
|  | **Consistently low (n=561)** | **Consistently moderate (n=402)** | **High but bell-shaped (n=60)** | ***P*** |  | **Consistently low (n=621)** | **Consistently moderate (n=321)** | **High but bell-shaped (n=81)** | ***P*** |
| Month 1 | 363 (67.0) | 254 (66.7) | 35 (60.3) | 0.593 |  | 402 (66.9) | 200 (66.4) | 50 (63.3) | 0.817 |
| Month 3 | 338 (65.4) | 237 (65.5) | 34 (57.6) | 0.479 |  | 380 (66.5) | 181 (62.6) | 48 (61.5) | 0.422 |
| Month 6 | 375 (76.1) | 243 (73.6) | 39 (72.2) | 0.656 |  | 413 (76.1) | 196 (73.7) | 48 (70.6) | 0.530 |
| Month 9 | 328 (71.5) | 229 (72.0) | 31 (67.4) | 0.810 |  | 369 (72.4) | 179 (70.2) | 40 (69.0) | 0.750 |
| Month 12 | 383 (77.2) | 262 (74.2) | 34 (61.8) | **0.038** |  | 422 (77.3) | 207 (72.9) | 50 (67.6) | 0.111 |

**Table S3** China Real-World Oral Intake of PrEP (CROPrEP) study team

| **First Name**  **and Middle Initial(s)** | **Last Name** | **Institution** |
| --- | --- | --- |
| Rui | Li | The First Affiliated Hospital of China Medical University |
| Qiang | Kang | The First Affiliated Hospital of China Medical University |
| Shangcao | Li | The First Affiliated Hospital of China Medical University |
| Zhili | Hu | The First Affiliated Hospital of China Medical University |
| Rantong | Bao | The First Affiliated Hospital of China Medical University |
| Hang | Li | The First Affiliated Hospital of China Medical University |
| Yonghui | Zhang | The First Affiliated Hospital of China Medical University |
| Zhu | Mei | The First Affiliated Hospital of China Medical University |
| Yueru | Jia | The First Affiliated Hospital of China Medical University |
| Zehao | Ye | The First Affiliated Hospital of China Medical University |
| Yanni | Ma | The First Affiliated Hospital of China Medical University |
| Xin | Ma | The First Affiliated Hospital of China Medical University |
| Xiaoyun | Shi | The First Affiliated Hospital of China Medical University |
| Yijun | Duan | Beijing Youan Hospital |
| Guanghui | Zhang | Beijing Youan Hospital |
| Xiaojie | Huang | Beijing Youan Hospital |
| Fang | Zhao | Shenzhen Third People's Hospital |
| Hui | Wang | Shenzhen Third People's Hospital |
| Lukun | Zhang | Shenzhen Third People's Hospital |
| Jin | Zhao | Shenzhen Center for Disease Control and Prevention |
| Yao | Li | Chongqing Public Health Medical Center |
| Yaokai | Chen | Chongqing Public Health Medical Center |
| Xiaoqing | He | Chongqing Public Health Medical Center |

**Table S4** Scores of PrEP adherence at different follow-up for each anxiety and depression trajectory

| **Variable** | **Follow-up point** | **M1**  **(median, quartile)** | **M3**  **(median, quartile)** | **M6**  **(median, quartile)** | **M9**  **(median, quartile)** | **M12**  **(median, quartile)** |
| --- | --- | --- | --- | --- | --- | --- |
| Anxiety | Consistently low | 1.00  (0.79, 1.00) | 0.98  (0.77, 1.00) | 1.00  (0.91, 1.00) | 1.00  (0.86, 1.00) | 1.00  (0.93, 1.00) |
|  | Consistently moderate | 1.00  (0.78, 1.00) | 0.98  (0.75, 1.00) | 1.00  (0.88, 1.00) | 1.00  (0.84, 1.00) | 1.00  (0.87, 1.00) |
|  | High but bell-shaped | 0.97  (0.79, 1.00) | 0.95  (0.70, 1.00) | 1.00  (0.85, 1.00) | 0.98  (0.83, 1.00) | 1.00  (0.78, 1.00) |
| Depression | Consistently low | 1.00  (0.79, 1.00) | 0.99  (0.78, 1.00) | 1.00  (0.91, 1.00) | 1.00  (0.87, 1.00) | 1.00  (0.94, 1.00) |
|  | Consistently moderate | 1.00  (0.78, 1.00) | 0.97  (0.74, 1.00) | 1.00  (0.88, 1.00) | 1.00  (0.84, 1.00) | 1.00  (0.85, 1.00) |
|  | High but bell-shaped | 1.00  (0.83, 1.00) | 0.98  (0.73, 1.00) | 1.00  (0.79, 1.00) | 0.99  (0.83, 1.00) | 1.00  (0.84, 1.00) |

Abbreviations: PrEP, Pre-Exposure Prophylaxis

**Table S5** Fitting models for various PrEP adherence trajectories

| **Subgroup number** | **BIC** | **AvePP (95% CI)** | | | | |
| --- | --- | --- | --- | --- | --- | --- |
|  |  | **1** | **2** | **3** | **4** | **5** |
| 1 | -849.76 | 1.00  (1.00-1.00) | NA | NA | NA | NA |
| 2 | -1874.84 | 0.96  (0.95-0.96) | 0.93  (0.92-0.95) | NA | NA | NA |
| 3 | -2260.32 | 0.90  (0.88-0.93) | 0.97  (0.96-0.97) | 0.89  (0.87-0.91) | NA | NA |
| 4 | -2326.20 | 0.91  (0.89-0.93) | 0.97  (0.96-0.98) | 0.89  (0.86-0.93) | 0.89  (0.87-0.92) | NA |
| 5 | -2291.66 | NA | 0.88  (0.85-0.91) | 0.88  (0.84-0.91) | 0.50  (0.50-0.50) | 0.89  (0.86-0.92) |

Abbreviations: PrEP, Pre-Exposure Prophylaxis; BIC, Bayesian Information Criterion; AvePP, Average Posterior Probability; CI, Confidence Interval; NA, not applicable


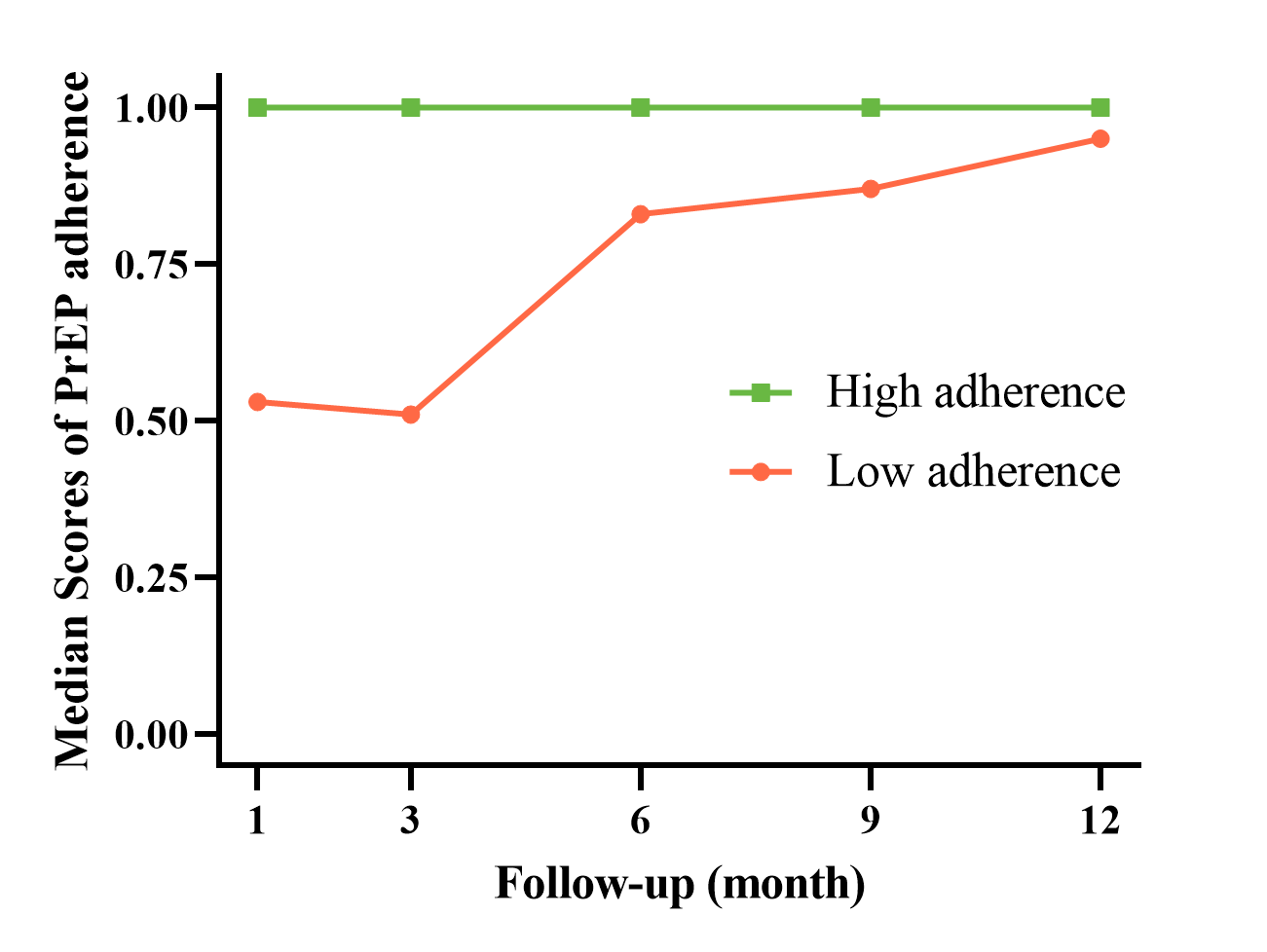


**Fig. S1** Trajectory profiles for PrEP adherence scores.

Abbreviations: PrEP, Pre-exposure prophylaxis

**Table S6** Association between different trajectories of anxiety and depression and trajectories of PrEP adherence scores

| **Variable** | **Adherence** | **High adherence** | **Low adherence** | ***P*** |
| --- | --- | --- | --- | --- |
| Anxiety | Consistently low | 398 (72.0) ^a^ | 155 (28.0) ^a^ | 0.926 |
|  | Consistently moderate | 274 (70.8) ^a^ | 113 (29.2) ^a^ |  |
|  | High but bell-shaped | 43 (71.7) ^a^ | 17 (28.3) ^a^ |  |
| Depression | Consistently low | 441 (72.2) ^a^ | 170 (27.8) ^a^ | 0.217 |
|  | Consistently moderate | 211 (68.5) ^a^ | 97 (31.5) ^a^ |  |
|  | High but bell-shaped | 63 (77.8) ^a^ | 18 (22.2) ^a^ |  |

^a^: Correcting using the Bonferroni. The same letter indicates that there is no statistical difference in the proportion between the two sub-groups

**Table S7** Participants initiating PrEP with HIV seroconversion during the study period

| **No.** | **PrEP regimen** | **Last HIV negative date** | **First HIV positive date** | **Days between Last negative and first positive date** | **Anxiety trajectories** | **Depression trajectories** | **Adherence scores** | **Self-reported medication adherence** |
| --- | --- | --- | --- | --- | --- | --- | --- | --- |
| 1 | D-PrEP | 2019/7/10 | 2019/8/5 | 26 | Consistently low | Consistently low | 0.00 | Reported condomless receptive anal intercourse with casual sex partner five days after stop taking PrEP |
| 2 | D-PrEP | 2019/10/31 | 2020/1/13 | 74 | Consistently low | Consistently low | 1.00 | Self-reported missing doses in 30 days and 10 sexual acts uncovered by PrEP in the past three months |
| 3 | D-PrEP | 2019/4/26 | 2019/6/19 | 54 | Consistently low | Consistently moderate | 0.65 | Follow-up was delayed for one month, intermittent medication, self-reported missing doses in 30 days and have 15 condomless receptive in the past two months |
| 4 | D-PrEP | 2019/8/19 | 2019/10/28 | 70 | Consistently moderate | Consistently low | 0.97 | Self-reported missing doses in 10 days and two sexual acts uncovered by PrEP in the past three months |
| 5 | D-PrEP | 2020/1/10 | 2020/8/7 | 210 | Consistently low | Consistently low | 0.43 | Self-reported missing doses in 90 days and more than 20 sexual acts uncovered by PrEP in the past three months |
| 6 | ED-PrEP | 2019/12/5 | 2020/5/22 | 169 | Consistently moderate | Consistently moderate | 1.00 | Self-reported missing doses in 25 days and three sexual acts uncovered by PrEP in the past three months |
| 7 | ED-PrEP | 2019/5/19 | 2019/6/22 | 34 | Consistently moderate | Consistently moderate | 0.25 | Reported 9 episodes of sexual behaviors after enrollment, missed 6 doses of PrEP before sexual behaviors, no missing doses 24h and 48h after sexual behaviors |

Abbreviations: PrEP, Pre-Exposure Prophylaxis; D-PrEP, Daily PrEP; ED-PrEP, Event-Driven PrEP
